# Tunable Non-Markovianity for Bosonic Quantum Memristors

**DOI:** 10.3390/e25050756

**Published:** 2023-05-06

**Authors:** Jia-Liang Tang, Gabriel Alvarado Barrios, Enrique Solano, Francisco Albarrán-Arriagada

**Affiliations:** 1 International Center of Quantum Artificial Intelligence for Science and Technology (QuArtist), Physics Department, Shanghai University, Shanghai 200444, China; 2 Kipu Quantum, Greifswalderstrasse 226, 10405 Berlin, Germany; 3 Departamento de Física, Universidad de Santiago de Chile (USACH), Avenida Víctor Jara 3493, Santiago 9170124, Chile; 4 Center for the Development of Nanoscience and Nanotechnology, Estación Central 9170124, Chile

**Keywords:** open quantum system, non-Markovanity, quantum memristor

## Abstract

We studied the tunable control of the non-Markovianity of a bosonic mode due to its coupling to a set of auxiliary qubits, both embedded in a thermal reservoir. Specifically, we considered a single cavity mode coupled to auxiliary qubits described by the Tavis–Cummings model. As a figure of merit, we define the dynamical non-Markovianity as the tendency of a system to return to its initial state, instead of evolving monotonically to its steady state. We studied how this dynamical non-Markovianity can be manipulated in terms of the qubit frequency. We found that the control of the auxiliary systems affects the cavity dynamics as an effective time-dependent decay rate. Finally, we show how this tunable time-dependent decay rate can be tuned to engineer bosonic quantum memristors, involving memory effects that are fundamental for developing neuromorphic quantum technologies.

## 1. Introduction

In open quantum systems, the Markovian approximation is widely used due to its mathematical simplicity and because it provides a good description of the phenomenology observed in the lab. The Markovian approximation implies that the state of the reservoir is not correlated at different times, which can be interpreted as a memoryless bath [1,2]. Nonetheless, in several real-world systems and applications, memory effects play an important role and have to be explicitly accounted for, such as in biological systems [3,4,5], quantum metrology [6,7], quantum simulation [8,9], and quantum memdevices [10,11,12,13,14]. Therefore, the manipulation of non-Markovianity is an important step for the development of new technologies, where quantum memristive devices could particularly benefit the realization of neuromorphic quantum computing [15,16,17].

On the other hand, the definition and quantification of non-Markovianity in quantum systems is still an open question [18,19,20,21,22]. Nevertheless, there are two widely accepted cases in the scientific community. One is based on the distinguishability of the states of a quantum system [21] undergoing dissipative evolution. This definition takes into account that when a quantum system interacts with a Markovian environment, the system’s information will flow unidirectionally to the environment. States evolving under these conditions can only lose distinguishability during the dynamics. Therefore, we can distinguish non-Markovian behavior if, for a dissipative evolution, the states recover some of their distinguishability during certain time intervals. The second definition of non-Markovianity is based on the behavior of entanglement between the system and an auxiliary system [22]. If at the initial moment of the evolution the system is correlated to some auxiliary system, then these quantum correlations can only monotonically decrease in time if the system is coupled to a Markovian environment. Therefore, we identify non-Markovianity with cases where the quantum correlations do not decrease monotonically with time. Both of these definitions of non-Markovianity provide a method to quantify its degree and involve an optimization process over all possible initial states for the evolution.

Recently, non-Markovian dynamics have become an area of active research both in theory and experiment, driven by the wide interest in quantum technologies [23,24,25,26,27,28,29]. In experiments using an all-optical setup, the transitions between Markovian and non-Markovian regimes can be reached, controlling the information backflow of the system [25] as well as the observation of the so-called weak non-markovianity regime [26].

In this article, we focus on dynamical non-Markovianity (DnM), which means the degree of non-Markovianity presented in a given dynamic. Specifically, we will focus on a system composed of a cavity mode (main system) coupled to a set of qubits (auxiliary systems) described by the Tavis–Cummings (TC) model [30,31,32] embedded in a Markovian bath. We are interested in studying the DnM that arises in the main system dynamics by tracing the auxiliary qubits, creating a tunable bosonic quantum memristor. We will also explore how the DnM can be manipulated by external control over the auxiliary systems. We found that by tuning the energy gap of the set of qubits, we can simulate a time-dependent decay rate in the cavity going from a regime with maximal DnM to another with minimal DnM and Markovian evolution. This tunable dynamical non-Markovianity allows us to define variables that follow a memristive behavior, obtaining an experimentally feasible, scalable and general framework to implement switchable memory devices that are useful for neuromorphic quantum computing.

## 2. Model and Methods

**Figure 1:**
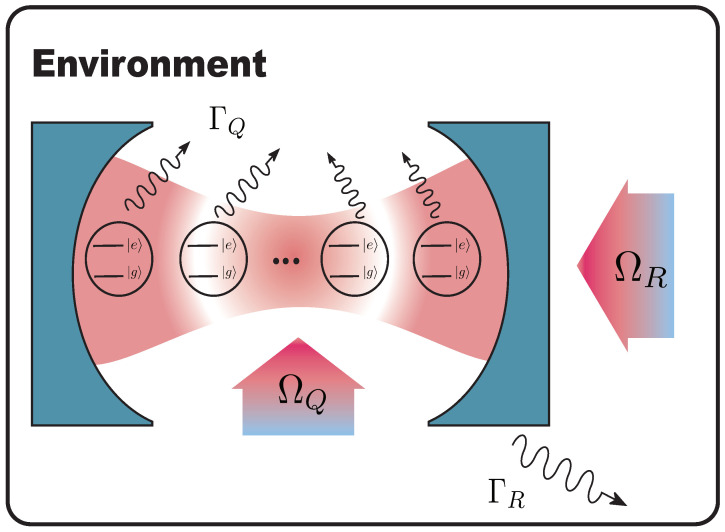
Diagram of the model: a cavity (bosonic mode) coupled to a set of qubits embedded in a Markovian reservoir. Each auxiliary qubit can be dynamically tuned, and the cavity can be classically driven.

We consider a system consisting of a single bosonic mode (resonator) coupled to a set of *n* qubits in contact with a thermal reservoir at zero temperature, as shown in Figure 1. The interaction between the qubits and the resonator is described by the TC model

(1) $${\hat{H}}_{TC}={\hat{H}}_{R}+{\hat{H}}_{Q}+{\hat{H}}_{R-Q}$$

where

(2) $$\begin{array}{ccc} {\hat{H}}_{R} & = & \hbar \omega_{R}{\hat{a}}^{†}\hat{a},\\ {\hat{H}}_{Q} & = & \frac{\hbar }{2}\sum_{j=1}^{n}\omega_{Q}{\hat{\sigma }}_{z,j},\\ {\hat{H}}_{R-Q} & = & \hbar \sum_{j=1}^{n}g\left({\hat{\sigma }}_{j}^{-}{\hat{a}}^{†}+{\hat{\sigma }}_{j}^{+}\hat{a}\right)), \end{array}$$

are the Hamiltonians for the bosonic mode, the qubits, and the resonator–qubits interaction, respectively. Here, $\omega_{R}$, $\omega_{Q}$, *g*, and *ℏ* denote the resonator frequency, the qubit frequency, the qubit-resonator coupling strength, and the Planck constant, respectively. The operator $\hat{a}\left({\hat{a}}^{†}\right)$ is the annihilation (creation) operator for the bosonic mode, ${\hat{\sigma }}_{z,j}$ is the Pauli *z* matrix for the *j*th qubit, and ${\hat{\sigma }}_{j}^{-(+)}$ is the lowering (raising) operator for the *j*th qubits. In order to ensure the validity of our model, we consider $\omega_{Q}/\omega_{R}∼1$ and $g/\omega_{R}<0.1$. From now on we will consider ℏ=1.

We consider that the total system undergoes Markovian dynamics described by the following master equation,

(3) $$\begin{array}{ccc} & & \dot{\rho }\left(t\right)=-i\left[{\hat{H}}_{TC},\rho \left(t\right)\right]+\sum_{j=0}^{n}\Gamma_{j}D\left[{\hat{O}}_{j}\right]\rho , \end{array}$$

with

(4) $$D\left[{\hat{O}}_{j}\right]\rho ={\hat{O}}_{j}\rho {\hat{O}}_{j}^{†}-\frac{1}{2}\left\{{\hat{O}}_{j}^{†}{\hat{O}}_{j},\rho \right\},$$

where ${\hat{O}}_{0}=a$ and ${\hat{O}}_{j}=\sigma_{j}^{-}$ for j>0 and $\Gamma_{j}$ is the decay rate of the *j*th channel. We are interested only in the dynamics of the resonator; thus, we focus on its reduced state by tracing out the qubits, $\rho_{R}\left(t\right)={\mathrm{Tr}}_{Q}\left(\rho \left(t\right)\right)$. In this way, the set of qubits act as an auxiliary system that introduces non-Markovian properties to the dissipative evolution of the resonator. We remark that the TC model has been experimentally realized in several platforms, such as quantum dots [33], trapped ions [34], and superconducting circuits [35]. We highlight that in superconducting circuits, it has been achieved up to 10 qubits coupled to a single resonator, which is even higher than the number of qubits we consider in this work to induce non-Markovian dynamics in the resonator.

We want to characterize the degree of non-Markovianity of a particular evolution of our system (the resonator) determined by its initial state. We look for a figure of merit that can be understood as a degree of non-Markovianity of the particular dynamics of the system that result from a given initial condition. To this end, we notice that when the dynamics of the system are Markovian and purely dissipative, then its quantum state will monotonically approach the corresponding steady state of the environment. We can characterize this behavior by calculating the trace distance between the instantaneous state of the system and the steady state of the evolution,

(5) $$D_{S}\left(\rho_{R}\left(t\right)\right)=\frac{1}{2}\left|\rho_{R}\left(t\right)-\rho_{SS}\right|,$$

where the subindex *S* denotes that the trace distance is taken with respect to the steady state. For a Markovian evolution, this quantity will decrease monotonically to zero [21], where $\left|\rho |=\mathrm{Tr}[\sqrt{\rho^{†}\rho }\right]$ and $\rho_{SS}$ is the steady state of the system. In our case, the temperature of the environment is zero, and therefore, $\rho_{SS}=\left|0\rangle \langle 0\right|$. Now, the quantity D(t) allows us to detect when the evolution deviates from Markovian behavior whenever it is no longer monotonically decreasing. Therefore, we can characterize the non-Markovianity of a particular system evolution by considering all the time intervals with non-monotonic behavior. In this way, we define the DnM as

(6) $$N_{D}=\int_{\zeta >0}\zeta \left(t\right)dt,$$

where $\zeta \left(t\right)=\left(d/dt\right)D_{S}\left(\rho_{R}\left(t\right)\right)$, and the integration time goes from 0 to infinite, which means until a sufficiently long time as the system reaches the steady state. Notice that for a given interval $t\in [\tau_{i},\tau_{j}]$, in which the trace distance detects information backflow (derivative is positive), the DnM over that interval is simply $D_{S}\left(\rho_{R}\left(\tau_{j}\right)\right)-D_{S}\left(\rho_{R}\left(\tau_{i}\right)\right)$, which provides a simple way of calculating $N_{D}$. In addition, this definition is closely related to the non-Markovianity measure for dissipative channels based on distinguishability [21]. However, our definition considers only the dynamics under study and not an optimization over all the initial conditions. We emphasize that the evolution of the total system is Markovian, and thus, it ultimately reaches the thermal steady state corresponding to the temperature considered. Nonetheless, the reduced dynamics of the resonator will be non-Markovian since the qubit–resonator coupling will introduce an information backflow from the set of qubits. In the next section, we will characterize how a given configuration of the set of qubits affects the behavior of the DnM for such a bosonic quantum memristor.

## 3. Results

### 3.1. Dynamical Non-Markovianity (DnM)

**Figure 2:**
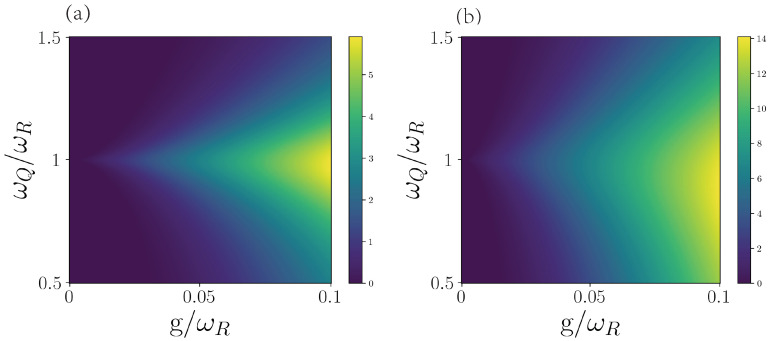
Dynamical non-Markovainity of the resonator. (**a**) One-qubit case. (**b**) Five-qubit case. In both cases, the decay rate of qubit and resonator is $\Gamma_{Q}=\Gamma_{R}=0.005$. We consider resonator frequency $\omega_{R}=1$, qubit frequency $\omega_{Q}/\omega_{R}\in \left[0.5,1.5\right]$, the coupling strength $g/\omega_{R}\in \left[0,0.1\right]$ and the initial state $|\psi_{0}\rangle =|1_{R}0_{Q}\rangle$.

For our first case, we will focus on the resonator interacting with one qubit (Jaynes–Cummings model) and interacting with n=5 qubits. We will analyze how the DnM depends on the qubit frequency and coupling strength. It is important to mention that the set of auxiliary qubits is always initialized in the ground state in order not to introduce energy into the resonator, since it would undermine the interpretation of the DnM. For our calculations, we numerically solve the master equation of Equation (3) in Python using the mesolve method in QuTiP [36]. Then, the DnM is calculated from the reduced state of the resonator. First, we consider the initial state $|\psi_{0}\rangle =|1_{R}0_{Q}\rangle$. In Figure 2a, we show the DnM of the resonator when varying the coupling strength $g/\omega_{R}$ and the frequency ratio $\omega_{Q}/\omega_{R}$. We can see that the DnM is largest when the qubit and resonator are in resonance and when *g* increases. Notice that for larger values of $g/\omega_{R}$, the qubit–resonator detuning can yield significant values of DnM. Figure 2b shows the DnM for the case of five auxiliary qubits. We can observe that the effect of enlarging the set of auxiliary qubits is relaxing the resonance condition and increasing the value for the DnM.

This behavior is to be expected since the resonance condition allows for maximal information transfer and information backflow due to the complete Rabi oscillations (in the case of n=1). In addition, the coupling strength $g/\omega_{R}$ is related to the speed of the information transfer and the information backflow. Then, for a small *g* (slow information transfer), a stronger resonance condition is needed to have information backflow before the system reaches the stationary state. If *g* increases, the communication between the auxiliary qubits and the bosonic mode is faster, and a more relaxed resonance condition will still have information backflow. Increasing the number of qubits increases the channels for information backflow, which leads to larger values of DnM even at higher detuning.

**Figure 3:**
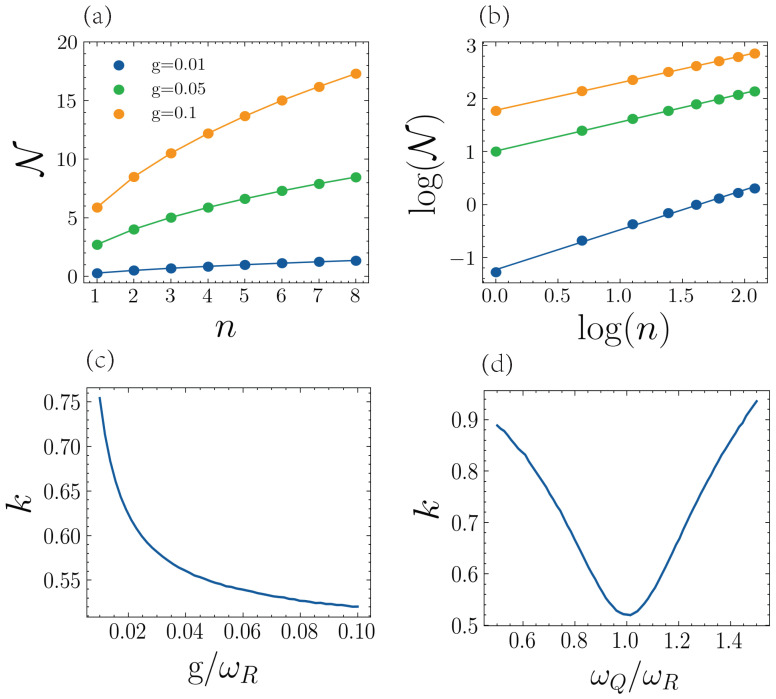
(**a**) The DnM of the resonator in terms of the number of qubits *n*. (**b**) The log–log plot of DnM and the number of qubits. For (**a**,**b**), we consider three cases, g=0.01,0.05,0.1, with the resonant condition $\omega_{Q}=\omega_{R}=1$. (**c**) The exponent *k* of the power–law dependence as a function of the coupling strength $g/\omega_{R}$, with qubit and resonator in resonance. (**d**) The exponent *k* of the power–law dependence as a function of the frequency of the qubit $\omega_{Q}/\omega_{R}$ and a fixed coupling strength $g/\omega_{R}=0.05$. For all cases, we consider decay rates $\Gamma_{Q}=\Gamma_{R}=0.005\omega_{R}$, the initial state of the resonator $|\psi_{0}\rangle =|1_{R}\rangle$, and all the qubits initialized in the ground state.

Next, we study the scaling of the DnM with the number of qubits under fixed conditions. In Figure 3, we study $N_{D}$ as a function of the number of qubits (until n=8) for different coupling strengths when the resonator is initialized with one excitation. Figure 3a shows how $N_{D}$ scales with the number of particles (*n*), with a monotonically increasing behavior reminiscent of a power law. In Figure 3b, we do a log–log plot of the quantities of Figure 3a, which confirms the power–law dependence. We perform a linear fit of the data in Figure 3b and obtain in all instances an $R^{2}$ coefficient larger than 0.995, which allows us to approximate the scaling of the DnM by

(7) $$N_{D}\propto n^{k},$$

where the exponent *k* depends on the coupling strength $g/\omega_{R}$ and the qubit frequency $\omega_{Q}/\omega_{R}$. In Figure 3c,d, we show the dependence of the exponent *k* on the coupling strength and the qubit frequency, respectively, which has been numerically calculated by the same fitting procedure as with Figure 3b. We can see that when the DnM is maximal, that is, for a large *g* and $\omega_{Q}=\omega_{R}$, the value of *k* is at a minimum.

The monotonically increasing behavior of the DnM obtained in Equation (7) is due to the frequency of the Rabi oscillations between the cavity and the set of qubits, which increases with the number of qubits and is caused by the collective enhancement of the coupling strength of the Tavis–Cummings model with *n* qubits [30]. This phenomenon behaves effectively as a cavity interacting with a single qubit with coupling strength $\sqrt{n}g$. Therefore, with faster Rabi oscillations, there are more time intervals where the trace distance is positive from the information backflow and yields a higher value of DnM. Increasing the number of qubits also reduces the relaxation time, and the initial excitation is dissipated faster, but with the parameters we have considered, increasing the number of qubits increases the Rabi oscillations faster than the relaxation. Thus, we obtain the behavior of Equation (7). We need to mention that as the Hilbert space of our system grows exponentially with the number of qubits, we can only calculate Figure 3a until eight qubits. Nevertheless, it is interesting in that it provides more numerical evidence of the power–law dependence given in Equation (7). In addition, an analysis of the thermodynamic limit of the proof of Equation (7) for a large qubit limit would be interesting but is out of the scope of the present study

We have seen that the DnM of the resonator strongly depends on the parameters of the set of auxiliary qubits. It is then interesting to consider whether we can have dynamic control over the DnM by manipulating the set of auxiliary qubits. In what follows, we apply a driving term in the *z*-direction to the set of auxiliary qubits in order to dynamically modulate the qubit gap and control the degree of DnM in the evolution. The driving is chosen so that it does not introduce energy into the qubits, which could excite the resonator and be interpreted as information backflow by the DnM. This situation is described by the following Hamiltonian:

(8) $$\hat{H}=H_{TC}+\hbar \Omega_{Q}\sum_{j}^{n}\sin\left(\mu_{Q}t\right){\hat{\sigma }}_{j}^{z},$$

where $H_{TC}$ is the Hamiltonian of Equation (1), and $\Omega_{Q}$ and $\mu_{Q}$ are the amplitude and frequency of the driving over the qubits, respectively. Notice that we consider that each qubit is driven by the same signal.

**Figure 4:**
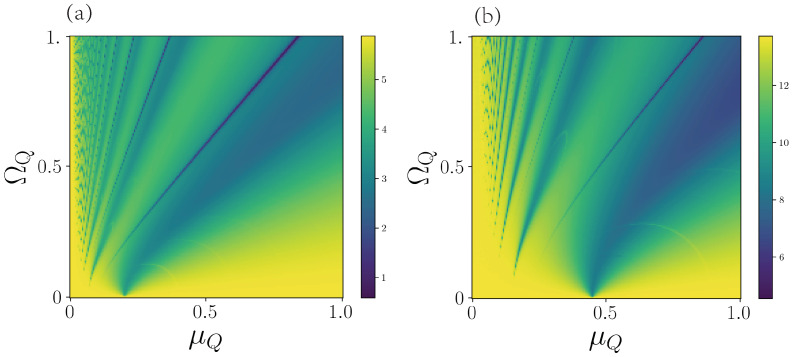
The non-Markovainity of the resonator. (**a**) One-qubit case. (**b**) Five-qubit case. Parameters: In both cases, the decaying rate of qubit and resonator is $\Gamma_{Q}=\Gamma_{R}=0.005\omega_{R}$. The driving frequency of qubit $\mu_{Q}/\omega_{R}\in \left[0,1\right]$, the driving amplitude of the qubit $\Omega_{Q}/\omega_{R}\in \left[0,1\right]$, the qubit frequency $\omega_{Q}/\omega_{R}=1$, and the coupling strength $g/\omega_{R}=0.1$. The initial state is $|\psi_{0}\rangle =|1_{R}0_{Q}\rangle$.

We numerically calculate $N_{D}$ for different values of the driving frequency and amplitude, which we show in Figure 4. In Figure 4a, we show the case of one auxiliary qubit. Here, there is a non-zero DnM over the whole range of parameters. However, it is interesting to notice the dark lines that are spanned from near the origin, where the DnM is almost completely suppressed. A similar behavior occurs when we increase the number of qubits, as is shown in Figure 4b for the five qubits case, where the DnM is suppressed over thin lines in the frequency/amplitude plane. Although the suppression is not as strong as in the one-qubit case, these lines show a significant decrease in the DnM. This indicates that by modulating either the frequency or the amplitude of the driving, we can enhance or suppress the DnM of the resonator.

Such suppression of the DnM could be explained by the phenomena of coherent destruction of tunneling [37]. It can be understood as the suppression of coherent evolution between two states of a system due to a coherent driving. We note that we are considering a TC model with at most one excitation evolving only under an amplitude damping channel. In such a situation, our model can be described as a three-level system, where we only populate the states $|1_{R}\rangle |{\bar{0}}_{Q}\rangle$, $|0_{R}\rangle |{\bar{1}}_{Q}\rangle$ and $|0_{R}\rangle |{\bar{0}}_{Q}\rangle$, where the state $|{\bar{1}}_{Q}\rangle$ is the uniform superposition of one excitation in the set of qubits, and $|{\bar{0}}_{Q}\rangle$ is the ground state for all the qubits. It has been shown that coherent destruction of tunneling is presented in a three-level system [38], as well as for the Jaynes–Cummings model [39], which suggests that for a particular value of frequency $\mu_{q}$ and amplitude $\Omega_{q}$, the coherent information transfer between the set of qubits and the cavity is suppressed, and therefore, the DnM goes to zero.

**Figure 5:**
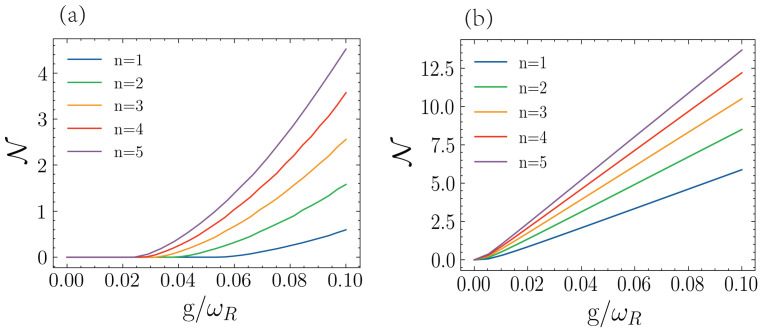
The minimum (**a**) and maximum (**b**) of DnM for the resonator with a different number of auxiliary qubits. In both cases, the decaying rate is $\Gamma_{Q}=\Gamma_{R}=0.005\omega_{R}$. The frequency of qubit $\omega_{Q}/\omega_{R}=1$, and the initial state is of resonator $|\psi_{0}\rangle =|1_{R}\rangle$. The qubits are all initialized in the ground state.

Similarly, we study the DnM in terms of the coupling strength for different numbers of qubits in the auxiliary system. Here, we will consider the driving parameters that yield the maximum and minimum DnM obtained from Figure 4 and the analogous calculations for n=2,3,4,5. In Figure 5a, we plot the minimal DnM for different coupling strengths *g*. We can see that up to g=0.02, we can essentially completely suppress the non-Markovian behavior by a suitable choice of driving parameters. Increasing the number of qubits decreases the necessary value of the coupling strength that allows for a completely suppressed DnM. On the other hand, in Figure 4b, we plot the maximal DnM for different coupling strengths *g*. Here, we can see that $N_{D}$ has a linear dependence on the coupling strength, except for a small range around zero. From these results, we have that, provided we choose a suitable value of the coupling strength, we can switch between Markovian and non-Markovian dynamics for the resonator just by controlling the auxiliary set of qubits.

**Figure 6:**
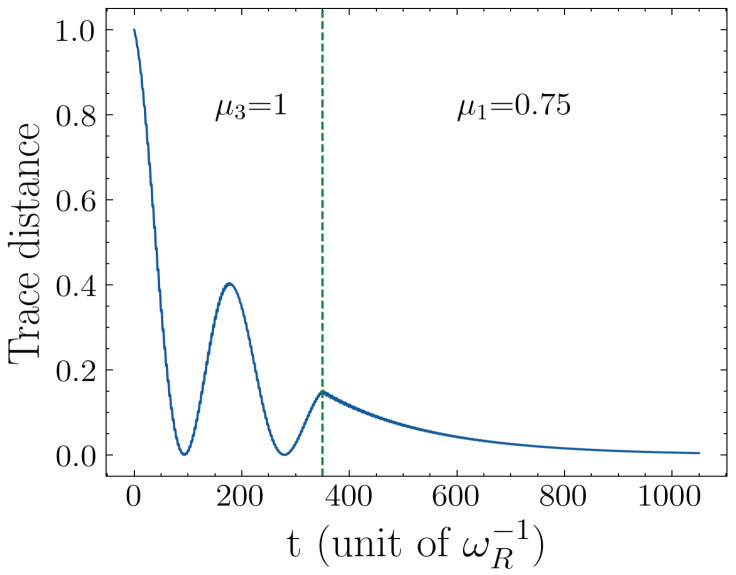
Transition from non-Markovian to Markovian dynamics by changing the driving frequency over the auxiliary qubits. Parameters: the driving amplitude of qubit is $\Omega_{q}=0.5$, the number qubit n=1, decaying rate is $\Gamma_{Q}=\Gamma_{R}=0.005$, frequency $\omega_{Q}=\omega_{R}$, and coupling strength g=0.05.

In Figure 6, we show how we can dynamically switch the non-Markovian behavior on and off just by changing the driving frequency of the qubits. Here, we plot the trace distance as a function of time. At the start of the evolution, we choose a driving frequency that yields maximum non-Markovianity ($\mu_{Q}=\omega_{R}$); later, at $t=350\omega_{R}^{-1}$, we switch the driving frequency to $\mu_{Q}=0.75\omega_{R}$, which yields the minimum DnM. As can be seen in the figure, at $t=350\omega_{R}^{-1}$, the trace distance switches from non-monotonic to monotonically decreasing behavior, which characterizes Markovian evolution.

**Figure 7:**
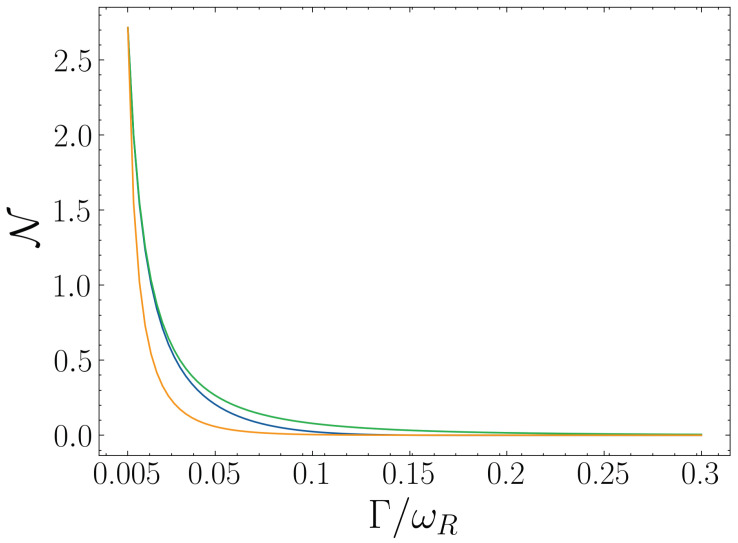
Dynamical non-Markovainity of the resonator varies with decaying rate. In the blue line, the decay rate of resonator $\Gamma_{R}=0.005$, and qubit decay rate $\Gamma_{Q}=\Gamma$. In the green line, the decay rate of qubit $\Gamma_{Q}=0.005$, and resonator decay rate $\Gamma_{R}=\Gamma$. In the orange line, the decay rate of qubit and resonator are the same, $\Gamma_{Q}=\Gamma_{R}=\Gamma$. We consider that qubit and resonator are in resonance ($\omega_{Q}=\omega_{R}=1$), the coupling strength $g/\omega_{R}=0.05$, and the initial state $|\psi_{0}\rangle =|1_{R}0_{Q}\rangle$.

Finally, we analyze the effect of the decay rates on the dynamical non-Markovianity. We consider varying the decay rates in three cases: varying it only for the qubits, only for the resonator, or varying both simultaneously. We plot the three cases in Figure 7; in general, we can see that smaller decay rates will yield larger values of dynamical non-Markovianity since the original excitation will survive longer in the system before being dissipated. Fixing all the other parameters, in all three cases considered, the DnM appears to exponentially decay as the decay rate increases, with the strongest effect being the case where the qubits and the cavity decay rate are changed simultaneously.

### 3.2. Time-Dependent Decay Rate

The observed memory effect can be understood as the system effectively interacting with an environment with a time-dependent decay rate, which becomes negative during some time intervals, favoring the information backflow [40]. To understand this statement, consider a resonator with state $\tilde{\rho }$ undergoing dissipative dynamics with a time-dependent decay rate and without any interaction with an auxiliary system; the system is then described by the following master equation:

(9) $$\dot{\tilde{\rho }}\left(t\right)=-i\left[H,\tilde{\rho }\right]+\Gamma \left(t\right)\left(a\tilde{\rho }a^{†}-\frac{1}{2}\left\{\tilde{\rho },a^{†}a\right\}\right)$$

where $H=\hbar \omega_{R}a^{†}a$, and Γ(t) is a time-dependent decay rate that can be negative. Here, $\tilde{\rho }$ represents the state of the resonator undergoing dynamics as described above and is different from $\rho_{R}$, which is the reduced state of the resonator as described by Hamiltonian (1). For Γ(t)>0, the energy of the resonator dissipates to the environment, meaning that the information in the resonator is continuously lost. Meanwhile, for Γ(t)<0, there is energy entering the resonator, giving way to information backflow and therefore to a non-Markovian process.

**Figure 8:**
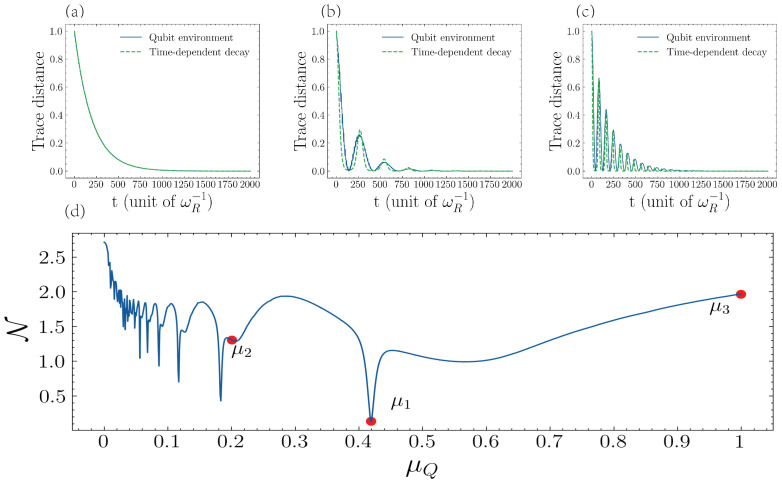
Trace distance of the resonator and the DnM under different driving frequencies. Top (**a**), the blue line—the frequency of driving $\mu_{1}=0.419$, the green line—the resonator’s decaying rate is constant Γ(t)=0.005. (**b**) The blue line—the frequency of driving $\mu_{2}=0.20$, the green line—the resonator’s decaying rate is $\Gamma_{1}\left(t\right)=0.05\left(\sin(0.023t)+0.09\right)$. (**c**) The blue line—the frequency of driving $\mu_{3}=1$. The green line—the resonator’s decaying rate is $\Gamma_{1}\left(t\right)=0.25\left(\sin(0.079t)+0.021\right)$. Bottom (**d**), the DnM of resonator in different driving frequencies $\mu_{Q}\in \left(0,1\right)$. Parameters: the number qubit n=1, decaying rate is $\Gamma_{Q}=\Gamma_{R}=0.005$, frequency $\omega_{Q}=\omega_{R}$, coupling strength g=0.05, and driving amplitude $\Omega_{Q}=0.5\omega_{R}$.

We consider the time-dependent decay rate parametrized as $\Gamma_{1}\left(t\right)=A\left(\sin(Bt)+C\right)$. Notice that the master equation in Equation (9) has the same steady state as that of our original system in Equation (3). Therefore, for a given dynamic induced by the set of auxiliary qubits, we can find the closest non-Markovian dynamic corresponding to a negative decay rate by finding *A*, *B*, and *C* that optimize the cost function $\int |D\left(\rho_{R}\left(t\right)\right)-D\left(\tilde{\rho }\left(t\right)\right){|}^{2}$, where $\tilde{\rho }\left(t\right)$ is the density operator calculated using the time-dependent decay rate. In Figure 8, we plot $D_{S}\left(\rho_{R}\left(t\right)\right)$ and $D_{S}\left({\tilde{\rho }}_{\mathrm{opt}}\left(t\right)\right)$, where $\rho_{R}\left(t\right)$ is for one qubit case and ${\tilde{\rho }}_{\mathrm{opt}}\left(t\right)$ is the resonator evolved with the optimal parameters for the decay rate.

We consider three cases:in Figure 8a, the effective decay rate is time-independent Γ=0.005, corresponding to Markovian behavior, whereas for the time-dependent decay rate we have in Figure 8b is Γ(t)=0.05[sin(0.023t)+0.09] and in Figure 8c it is Γ(t)=0.25[sin(0.079t)+0.021]. Finally, Figure 8d shows the DnM as a function of the qubit-driving frequency, where it displays the qubit-driving frequency corresponding to Figure 8a–c. We can see that for time-dependent decay, the behavior of both trace distance is almost the same, which means that the set of auxiliary qubits can switch on/off highly non-Markovian dynamics.

Finally, it is interesting to study how this simulated time-dependent decay rate can affect the response of the cavity over external driving, in order to control the memristive properties of the dynamics.

### 3.3. Bosonic Quantum Memristor

One interesting application of our results is to induce memristive behavior into the bosonic mode, which can be tuned by the set of auxiliary qubits. In Ref. [41], it was shown that a certain kind of time-dependent decay rate produces a quantum memristor, which could be reached in a superconducting circuits platform. Later, in Ref. [42], a memristive dynamic was obtained in a quantum computer by the simulation of a non-Markovian bath. In this line, we analyze the response of the cavity under an external driving, obtaining a Hamiltonian of the form

(10) $$\hat{H}=H_{TC}+\hbar \Omega_{Q}\sum_{j}^{n}\sin\left(\mu_{Q}t\right){\hat{\sigma }}_{j}^{z}+F\left(t\right)\left(a+a^{†}\right).$$

Now, we define the variables $I=-\langle i\left(a-a^{†}\right)\rangle$ and $O=\langle \dot{N}\rangle +\alpha \langle N\rangle$ with α a constant. If we consider $\alpha =\Gamma_{c}$ as the natural decay rate of the cavity, we have that

(11) O=F(t)I+G(t),

and for more details see Appendix A. The function G(t) depends on the DnM of the system, which means that we can control the memristive relation

(12) O=F(t)I,

by controlling the value of G(t). Now, if we choose $F\left(t\right)=\Omega_{c}\left[1-\sin\left(\cos\mu_{c}t\right)\right]$, it is possible to obtain the typical pinched hysteresis loop that characterizes a quantum memristor.

**Figure 9:**
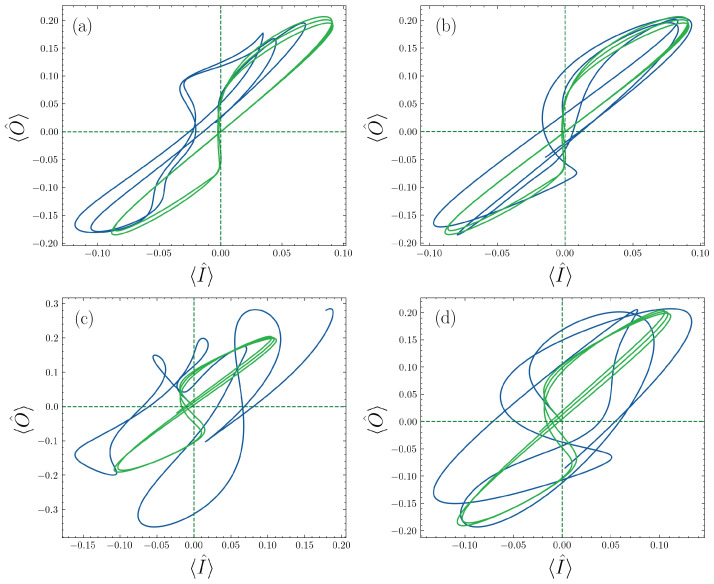
Memristive behavior; the green line shows the dynamics when the auxiliary qubits are not driven and off-resonant, and the blue curve is when we add a driving over the auxiliary qubits. (**a**) Larger-DnM case—the number of qubits n=1 and driving frequency $\mu_{c}=1$. (**b**) Medium-DnM case—the number of qubits n=1 and driving frequency $\mu_{c}=0.2$. (**c**) Larger-DnM case—the number of qubits n=5 and driving frequency $\mu_{c}=1$. (**d**) Medium-DnM case—the number of qubits n=5 and driving frequency $\mu_{c}=0.2$. Parameters: the driving amplitude of qubit is $\Omega_{q}=0.5$, decaying rate is $\Gamma_{Q}=\Gamma_{R}=0.005$, frequency $\omega_{Q}=\omega_{R}$, coupling strength g=0.05, the driving amplitude of cavity $\Omega_{c}=0.2$, and frequency $\mu_{c}=0.5$.

This situation is shown in Figure 9, where we obtain the pinched hysteresis loop (green curve) in a similar way as it has been obtained for previous proposals of quantum memristos [12,14,41] as a signature of memristive behavior. It is interesting to note that such bosonic quantum memristor dynamics appear when the auxiliary qubits are not driven and off-resonant with the cavity, which means that in an effective way, the qubits are decoupled from the cavity. In contrast, we can observe that when we drive the qubits, the memristive behavior can be destroyed for different cases, obtaining a way to go from memristive dynamics to non-memristive dynamics. It means that we also can control the memory properties induced by the decay rate in the cavity, which can be helpful in neuromorphic computing, considering that the proposed system can be implemented in many platforms such as optical devices, trapped ions, and superconducting circuits, among others. We also need to remark that our proposal can work as a switchable bosonic quantum memristor. This suggests that our formalism allows for implementing devices with controllable and switchable memory properties only by tuning the energy gap of auxiliary qubits. This proposal opens the door for the experimental implementation of memristive devices, providing a general, platform-free, and scalable model for the next generation of neuromorphic quantum computing technology.

In the present study, we have shown results for the homogeneous TC model, where all auxiliary qubits are identical in frequency, dissipation, and coupling with the cavity. The extension of our study to an inhomogeneous system is an interesting possibility for future research, which may focus on controlling the response of the cavity, opening the door to designing memristive devices with a given response. Nevertheless, the extension of our study to such disordered systems is computationally demanding and is left for future studies.

## 4. Conclusions

We have considered a cavity coupled to a set of auxiliary qubits, which induce a controllable dynamical non-Markovianity (DnM). We show that by the dynamical tuning of the energy gap of the auxiliary qubits, we can go from high to low values of DnM. The dynamics induced by the auxiliary qubits can be considered as an effective time-dependent and tunable decay rate. We also showed that the induced DnM in the cavity mode follows a power–law dependence with the number of auxiliary qubits, at least for a low number of qubits. Finally, we showed as an application that we can define memristive response in the cavity mode, which can be switched off by controlling the energy gap of the auxiliary qubits. This means that we can control the dynamical response of the cavity by external control of the auxiliary system, obtaining a switchable bosonic quantum memristor. These results provide a general protocol to obtain controllable bosonic quantum memristors, which can be useful in neuromorphic quantum computing. As our proposal only considers a Tavis–Cummings model where qubits are considered with a tunable energy gap, our work is experimentally feasible in different platforms, such as trapped ions and a superconducting circuit. Finally, the present work opens the door for the implementation of quantum memristors with minimal experimental requirements, paving the way for implementable neuromorphic quantum computing in the near future.

## Data Availability

The data in this article are available upon reasonable request from the corresponding author.

## Abbreviations

The following abbreviations are used in this manuscript:

DnM	Dynamical non-Markovianity

## Appendix A

The derivative of expectation for the number of photons $\langle {\hat{a}}^{†}\hat{a}\rangle$

(A1) $$\frac{d\langle {\hat{a}}^{†}\hat{a}\rangle }{dt}=\mathrm{Tr}\left[-\frac{i}{\hbar }\left[H,\rho \right]{\hat{a}}^{†}\hat{a}\right]+\mathrm{Tr}\left[L\left(\rho \right)a^{†}a\right]=S_{1}+S_{2}$$

we set ℏ=1, $S_{1}$ and $S_{2}$ is

(A2) $$\begin{array}{ccc} S_{1} & = & -i\mathrm{Tr}\left[\left[\omega_{c}a^{†}a+\omega_{q}\sum_{j=1}^{n}\sigma_{j}^{z}+\sum_{j=1}^{n}g\left(a^{†}\sigma_{j}+a\sigma_{j}^{+}\right)+\sum_{j=1}^{n}\Omega_{q}\sin\left(\mu_{q}t\right)\sigma_{j}^{z}+F\left(t\right)\left(a+a^{†}\right),\rho \right]{\hat{a}}^{†}\hat{a}\right]\\ & = & -i\mathrm{Tr}\left[\left(\sum_{j=1}^{n}g\left(a^{†}\sigma_{j}+a\sigma_{j}^{+}\right)\rho -\rho \sum_{j=1}^{n}g\left(a^{†}\sigma_{j}+a\sigma_{j}^{+}\right)+F\left(t\right)\left(a+a^{†}\right)\rho -\rho F\left(t\right)\left(a+a^{†}\right)\right){\hat{a}}^{†}\hat{a}\right]\\ & = & -i\mathrm{Tr}\left[\sum_{j=1}^{n}g\left(\sigma_{j}\rho a^{†}aa^{†}-\sigma_{j}^{+}\rho a^{†}aa-\rho a^{†}\sigma_{j}a^{†}a-\rho a\sigma_{j}^{+}a^{†}a\right)+F\left(t\right)\rho \left(\rho a^{†}a+\rho a^{†}aa^{†}a-\rho aa^{†}a-\rho a^{†}a^{†}a\right)\right]\\ & = & -i\mathrm{Tr}\left[\sum_{j=1}^{n}g\rho \left(\sigma^{+}\left(a^{†}a-aa^{†}\right)a+\sigma a^{†}\left(aa^{†}-a^{†}a\right)\right)+F\left(t\right)\rho \left(\left(a^{†}a-aa^{†}\right)a+a^{†}\left(aa^{†}-a^{†}a\right)\right)\right]\\ & = & -i\mathrm{Tr}\left[\sum_{j=1}^{n}g\rho \left(-\sigma^{+}a+\sigma a^{†}\right)+F\left(t\right)\rho \left(-a+a^{†}\right)\right]\\ & = & \langle \sum_{j=1}^{n}ig\left(-\sigma^{+}a+\sigma a^{†}\right)\rangle +F\left(t\right)\langle i(-a+a^{†})\rangle \\ & = & \sum_{j=1}^{n}g\langle i\left(-\sigma^{+}a+\sigma a^{†}\right)\rangle -F\left(t\right)\langle \hat{P}\rangle \end{array}$$

(A3) $$\begin{array}{ccc} S_{2} & = & \mathrm{Tr}\left[\Gamma_{c}\left(a\rho a^{†}-\frac{1}{2}\left(a^{†}a\rho +\rho a^{†}a\right)\right)a^{†}a+\Gamma_{q}\left(\sigma \rho \sigma^{+}-\frac{1}{2}\left(\sigma^{+}a+\rho \sigma^{+}\sigma \right)\right)a^{†}a\right]\\ & = & \mathrm{Tr}\left[\Gamma_{c}\rho \left(a^{†}a^{†}aa-\frac{1}{2}a^{†}aa^{†}a-\frac{1}{2}a^{†}aa^{†}a\right)\right]\;\\ & = & \mathrm{Tr}\left[\Gamma_{c}\rho \left(a^{†}\left(aa^{†}-1\right)a-\frac{1}{2}a^{†}aa^{†}a-\frac{1}{2}a^{†}aa^{†}a\right)\right]\;\\ & = & -\Gamma_{c}\langle (a^{†}a)\rangle \end{array}$$

so the derivative of expectation photon number is

(A4) $$\frac{d\langle {\hat{a}}^{†}\hat{a}\rangle }{dt}=\sum_{j=1}^{n}g\langle i\left(-\sigma^{+}a+\sigma a^{†}\right)\rangle -F\left(t\right)\langle \hat{P}\rangle -\Gamma_{c}\langle (a^{†}a)\rangle$$

The input and output are

(A5) $$\langle \hat{I}\rangle =-\langle \hat{P}\rangle$$

(A6) $$\langle \hat{O}\rangle =\frac{d\langle {\hat{a}}^{†}\hat{a}\rangle }{dt}+\Gamma_{c}\langle (a^{†}a)\rangle -G\left(t\right)$$

where $G\left(t\right)=\sum_{j=1}^{n}g\langle i\left(-\sigma^{+}a+\sigma a^{†}\right)\rangle$. As G(t) depends on the interaction between the qubits and the cavity, it can be controlled by the external driving over the set of auxiliary qubits. This means that G(t) will be close to zero when the qubits are off-resonance with the cavity, in which case we can neglect this term and write the output as

(A7) $$\langle \hat{O}\rangle =\frac{d\langle {\hat{a}}^{†}\hat{a}\rangle }{dt}+\Gamma_{c}\langle (a^{†}a)\rangle$$

The relation between input and output is

(A8) $$\hat{O}=F\left(t\right)\hat{I}$$

where F(t) is $\Omega_{c}\left((1-\sin\left(\cos\mu_{c}t\right)\right)$.

## References

[1] Li C.-F., Guo G.-C., Pillo J. (2019). Non-Markovian quantum dynamics: What does it mean?. EPL.

[2] Li C.-F., Guo G.-C., Pillo J. (2020). Non-Markovian quantum dynamics: What is it good for?. EPL.

[3] Lee H., Cheng Y.-C., Fleming G.R. (2007). Coherence Dynamics in Photosynthesis: Protein Protection of Excitonic Coherence. Science.

[4] Chin A.W., Datta A., Caruso F., Huelga S.F., Plenio M.B. (2010). Noise-assisted energy transfer in quantum networks and light-harvesting complexes?. New J. Phys..

[5] Fleming G.R., Huelga S.F., Plenio M.B. (2011). Focus on quantum effects and noise in biomolecules. New J. Phys..

[6] Alex W.C., Susana F.H., Martin B.P. (2012). Quantum Metrology in Non-Markovian Environments. Phys. Rev. Lett..

[7] Mirkin N., Larocca M., Wisniacki D. (2020). Quantum metrology in a non-Markovian quantum evolution. Phys. Rev. A.

[8] Sweke R., Sanz M., Sinayskiy I., Petruccione F., Solano E. (2016). Digital quantum simulation of many-body non-Markovian dynamics. Phys. Rev. A.

[9] Barreiro J.T., Müller M., Schindler P., Nigg D., Monz T., Chwalla M., Hennrich M., Roos C.F., Zoller P., Blatt R. (2011). An open-system quantum simulator with trapped ions. Nature.

[10] Pfeiffer P., Egusquiza I.L., Di Ventra M., Sanz M., Solano E. (2016). Quantum memristors. Sci. Rep..

[11] Spagnolo M., Morris J., Piacentini S., Antesberger M., Massa F., Crespi A., Ceccarelli F., Osellame R., Walther P. (2022). Experimental photonic quantum memristor. Nat. Photon..

[12] Sanz M., Lamata L., Solano E. (2018). Quantum memristors in quantum photonics. APL Photonics.

[13] Shevchenko S.N., Pershin Y.V., Nori F. (2016). Qubit-Based Memcapacitors and Meminductors. Phys. Rev. Appl..

[14] Norambuena S., Torres F., Di Ventra M., Coto R. (2022). Polariton-Based Quantum Memristors. Phys. Rev. Appl..

[15] Pershin Y.V., Di Ventra M. (2012). Neuromorphic quantum computing. Proc. IEEE.

[16] Pehle C., Wetterich C. (2022). Digital quantum simulation of many-body non-Markovian dynamics. Phys. Rev. E.

[17] Xu H., Krisn A.T., Verstraelen W., Liew T.C.H., Ghosh S. (2021). Superpolynomial quantum enhancement in polaritonic neuromorphic computing. Phys. Rev. B.

[18] De Vega I., Aloso D. (2017). Dynamics of non-Markovian open quantum systems. Rev. Mod. Phys..

[19] Rivas Á., FHuelga S., BPlenio M. (2014). Quantum non-Markovianity: Characterization, quantification and detection. Rep. Prog. Phys..

[20] Breuer H.P. (2012). Foundations and measures of quantum non-Markovianity. J. Phys. B: At. Mol. Opt. Phys..

[21] Breuer H.P., Laine E.M., Piilo J. (2009). Measure for the Degree of Non-Markovian Behavior of Quantum Processes in Open Systems. Phys. Rev. Lett..

[22] Rivas Á., Huelga S.F., Plenio M.B. (2010). Entanglement and Non-Markovianity of Quantum Evolutions. Phys. Rev. Lett..

[23] Luchnikov I.A., Vintskevich S.V., Grigoriev D.A., Filippov S.N. (2020). Machine Learning Non-Markovian Quantum Dynamics. Phys. Rev. Lett..

[24] Bastidas V.M., Kyaw T.H., Tangpanitanon J., Romero G., Kwek L.C., Angelakis D.G. (2018). Floquet stroboscopic divisibility in non-Markovian dynamics. New J. Phys..

[25] Liu B.-H., Li L., Huang Y.-F., Li C.-F., Guo G.-C., Laine E.-M., Breuer H.-P., Piilo J. (2011). Experimental control of the transition from Markovian to non-Markovian dynamics of open quantum systems. Nat. Phys..

[26] Bernardes N.K., Cuevas A., Orieux A., Monken C.H., Mataloni P., Sciarrino F., Santos M.F. (2015). Experimental observation of weak non-Markovianity. Sci. Rep..

[27] Li B.-W., Mei Q.-X., Wu Y.-K., Cai M.-L., Wang L., Yao L., Zhou Z.-C., Duan L.-M. (2022). Observation of Non-Markovian Spin Dynamics in a Jaynes-Cummings-Hubbard Model using a Trapped-Ion Quantum Simulator. Phys. Rev. Lett..

[28] García-Pérez G., Rossi M.A., Maniscalco S. (2020). IBM Q Experience as a versatile experimental testbed for simulating open quantum systems. NPJ Quantum Inf..

[29] Chen X.-Y., Zhang N.-N., He W.-T., Kong X.-Y., Tao M.-J., Deng F.-G., Ai Q., Long G.-L. (2022). Global correlation and local information flows in controllable non-Markovian open quantum dynamics. NPJ Quantum Inf..

[30] Tavis M., Cummings W. (1968). Exact Solution for an N-Molecule-radiation-Field Hamiltonian. Phys. Rev..

[31] Retzker A., Solano E., Reznik E. (2007). Tavis-Cummings model and collective multiqubit entanglement in trapped ions. Phys. Rev. A.

[32] Jäger S.B., Schmit T., Morigi G., Holl M.J., Betzholz R. (2022). Lindblad Master Equations for Quantum Systems Coupled to Dissipative Bosonic Modes. Phys. Rev. Lett..

[33] Van Woerkom D.J., Scarlino P., Ungerer J.H., Müller C., Koski J.V., Landig A.J., Reichl C., Wegscheider W., Ihn T., Ensslin K. (2018). Microwave Photon-Mediated Interactions between Semiconductor Qubits. Phys. Rev. X.

[34] Casabone B., Friebe K., Brandstätter B., Schüppert K., Blatt R., Northup T.E. (2020). Enhanced Quantum Interface with Collective Ion-Cavity Coupling. Phys. Rev. Lett..

[35] Wang Z., Li H.-K., Feng W., Song X.-H., Song C., Liu W.-X., Guo Q.-J., Zhang X., Dong H., Zheng D.-N. (2015). Controllable Switching between Superradiant and Subradiant States in a 10-qubit Superconducting Circuit. Phys. Rev. Lett..

[36] Johansson J., Nation P., Nori F. (2013). QuTiP 2: A Python framework for the dynamics of open quantum systems. Comput. Phys. Commun..

[37] Grossmann F., Dittrich T., Jung P., Hänggi P. (1991). Coherent destruction of tunneling. Phys. Rev. Lett..

[38] Neu P., Silbey R.J. (1996). Tunneling in a cavity. Phys. Rev. A.

[39] Luo X., Li L., You L., Wu B. (2014). Coherent destruction of tunneling and dark Floquet state. New J. Phys..

[40] Hu M.-L., Lian H.-L. (2015). Geometric quantum discord and non-Markovianity of structured reservoirs. Ann. Phys..

[41] Salmiletho J., Deppe F., Di Ventra M., Sanz M., Solano E. (2017). Quantum Memristors with Superconducting Circuits. Sci. Rep..

[42] Guo Y.-M., Albarrán-Arriagada F., Alaeian H., Solano E., Barrios G.A. (2022). Quantum Memristors with Quantum Computers. Phys. Rev. Appl..

